# Proliferation pattern during rostrum regeneration of the symbiotic flatworm *Paracatenula galateia*: a pulse-chase-pulse analysis

**DOI:** 10.1007/s00441-012-1426-4

**Published:** 2012-05-22

**Authors:** Ulrich Dirks, Harald R. Gruber-Vodicka, Bernhard Egger, Jörg A. Ott

**Affiliations:** 1 Department of Marine Biology, University of Vienna, Althanstrasse 14, 1090 Vienna, Austria; 2 Ultrastructural Research and Evolutionary Biology, Institute of Zoology, University of Innsbruck, Technikerstrasse 25, 6020 Innsbruck, Austria; 3 Department of Genetics, Evolution and Environment, University College London, Gower Street, Darwin Building, WC1E 6BT London, UK

**Keywords:** Morphallaxis, Epimorphosis, Bacteriocyte, Paratomy, Blastema, Neoblast

## Abstract

The remarkable totipotent stem-cell-based regeneration capacities of the Platyhelminthes have brought them into the focus of stem cell and regeneration research. Although selected platyhelminth groups are among the best-studied invertebrates, our data provide new insights into regenerative processes in the most basally branching group of the Platyhelminthes, the Catenulida. The mouth- and gutless free-living catenulid flatworm *Paracatenula galateia* harbors intracellular bacterial symbionts in its posterior body region, the trophosome region, accounting for up to 50% of the volume. Following decapitation of this flatworm, we have analyzed the behavior of the amputated fragments and any anterior and posterior regeneration. Using an EdU-pulse-chase/BrdU-pulse thymidine analog double-labeling approach combined with immunohistochemistry, we show that neoblasts are the main drivers of the regeneration processes. During anterior (rostrum) regeneration, EdU-pulse-chase-labeled cells aggregate inside the regenerating rostrum, whereas BrdU pulse-labeling before fixation indicates clusters of S-phase neoblasts at the same position. In parallel, serotonergic nerves reorganize and the brain regenerates. In completely regenerated animals, the original condition with S-phase neoblasts being restricted to the body region posterior to the brain is restored. In contrast, no posterior regeneration or growth of the trophosome region in anterior fragments cut a short distance posterior to the brain has been observed. Our data thus reveal interesting aspects of the cellular processes underlying the regeneration of the emerging catenulid-bacteria symbiosis model *P. galateia* and show that a neoblast stem cell system is indeed a plesiomorphic feature of basal platyhelminths.

## Introduction

From the human point of view, the capability to regenerate lost body parts is a fascinating phenomenon. Indeed, among many invertebrates, regeneration is a common feature, which is, in many cases, also connected with asexual reproduction. For more than 100 years, Platyhelminthes have acted as famous model systems for studying the principles underlying the regeneration of lost body parts. Because of their extraordinary regeneration capacity, some flatworms have been called “almost immortal under the edge of the knife” (Dalyell 1814). Today, we know of the huge variety of regenerative powers exhibited by the various flatworm taxa and researchers have tried to categorize and to determine a pattern explaining why some species can regenerate lost body parts and others cannot (for a review, see Egger et al. 2007). Comparative data suggest a link between asexual reproduction and regenerative capabilities, as these processes co-occur and show strong parallels in their progression pattern. Therefore, the use of the term “pregeneration” has been suggested as an alternative for those modes of asexual reproduction in which the differentiation of tissues occurs before the fragmentation (e.g., paratomy or budding). An essential feature for the regeneration processes shared by all Platyhelminthes is their remarkable system of pluripotent stem cells: the neoblast (Bode et al. 2006 and literature therein; Newmark and Sanchez Alvarado 2000). In all studied taxa, neoblasts are the only proliferating cells in adult flatworms and hence, they are the sole source of differentiated cell types not only during the continued cell-turnover but also during the regeneration of lost body parts. Moreover, neoblasts have been detected in the most basally branching group of the Platyhelminthes, the Catenulida and their importance for regeneration has been shown (Moraczewski 1977; Palmberg 1990).

In previous studies, we have investigated the way in which the mouth- and gutless catenulid flatworms of the genus *Paracatenula* reproduce asexually by paratomy and thus vertically transmit their intracellular “*Candidatus* Riegeria” bacterial symbionts to their asexual offspring (Dirks et al. 2012). We have described details of the neoblast system of *Paracatenula* and the capability of these worms to regenerate the rostrum after head amputation (Leisch et al. 2011). Since indications for sexual reproduction in this genus are rare and as this method of reproduction is not yet established, *Paracatenula* species are thought predominantly to reproduce asexually and thus maintain a permanent stock of their obligate intracellular symbionts. Gruber-Vodicka et al. (2011) have shown a tight co-diversification between the various hosts in the genus and their specific symbionts, further suggesting strict vertical symbiont transmission.

In the present study, we characterize morphological details and the role of neoblast stem cells during rostrum regeneration of *Paracatenula galateia* (Dirks et al. 2011). Using an EdU-pulse-chase and a BrdU-pulse approach, we have been able to selectively label and trace stem cells both before experimental decapitation and during the following regeneration process. This study is the first detailed investigation into cell proliferation patterns in regenerative processes of a symbiotic flatworm (*sensu strictu*).

## Materials and methods

### Sampling

Since none of the *Paracatenula* species can be grown in culture, all experiments that required live animals were performed immediately after sampling at the field laboratories in Carrie Bow Cay, Belize (16 °48′11 N, 88 °04′55 W) and Dahab, Egypt (28 °28′13.83″N, 34 °30′32.51″E). Sediments were collected in shallow water in the vicinity of Carrie Bow Cay (February 2009 and 2010) or in the Napoleon Reef in Dahab (June 2010). The worms were extracted by gently shaking the sand with ample amounts of filtered sea water (FSW) followed by pouring the supernatant through a 63-μm pore-sized mesh that retained the animals. Animals were then immediately washed from the mesh into Petri dishes and selected by hand with Pasteur pipettes under a dissecting microscope. They were then either fixed (see below) or kept alive for various experiments for up to 16 days in 2-ml glass vials containing FSW and a small amount of sediment from the sampling area.

### Decapitation and pulse-chase-pulse incubations

For the labeling of S-phase cells, the thymidine analogs EdU (5-ethynyl-2′-deoxyuridine, Click-it EdU Kit, Invitrogen) or BrdU (5-bromo-2′-deoxyuridine, Sigma) were dissolved in FSW to give a concentration of 2.5 mM. Animals were incubated for 30 min in EdU-containing FSW followed by five washes in FSW. Subsequently, they were reversibly anesthetized with MgCl_2_ solution isotonic to FSW and a razor blade was used to decapitate the animals transversally posterior to the brain region. The fragments were carefully placed into the prepared glass culture tubes and kept there for various chase times at room temperature (~25°C) under low lighting. At 24-h intervals, the animals were observed for up to a total of 384 h. At various stages of regeneration (chase times of 48 h, 120 h, 172 h, 264 h and 384 h; *n*=5 for each chase time), animals were subjected to a 30-min BrdU-pulse. The worms were then washed five times in FSW, briefly anesthetized with MgCl_2_ and fixed in 4% formaldehyde in phosphate-buffered saline (PBS) for 12 h at 4°C. Fixed animals were stored in pure methanol at -20°C for longer periods.

### Click chemistry and immunocytochemistry

Alexa fluor 488-azide fluorescent dye was covalently connected to the EdU-label in fixed specimens of *P. galateia* by performing “click reaction” following the protocol of the EdU click-iT Kit (Invitrogen). Staining with antibodies against BrdU (B&D) and serotonin (staining serotonergic nerves; Sigma) was performed according to the immunostaining protocol established by Ladurner et al. (1997, 2000), except for the protease treatment. We used Proteinase K (Sigma) at a final concentration of 0.1 mg/ml for up to 10 min at room temperature. Negative controls were conducted for both EdU-click-iT and all antibody staining. Fluorescently stained whole animals were mounted on slides and scanned with a confocal laser-scanning microscope (Zeiss LSM 510).

Further analysis and processing of the images was performed with a Zeiss LSM Image Browser and Adobe Photoshop CS5 software. Illustrations were produced with Adobe Illustrator CS5 software.

### Light microscopy

In the field laboratory, live animals were carefully squeezed under a coverslip on a microscope slide and observed with a phase contrast microscope (Zeiss, Germany). Live animals were transferred back to their culture dishes after observation and thus, the same specimen could be observed at various time intervals.

## Results

**Fig. 1:**
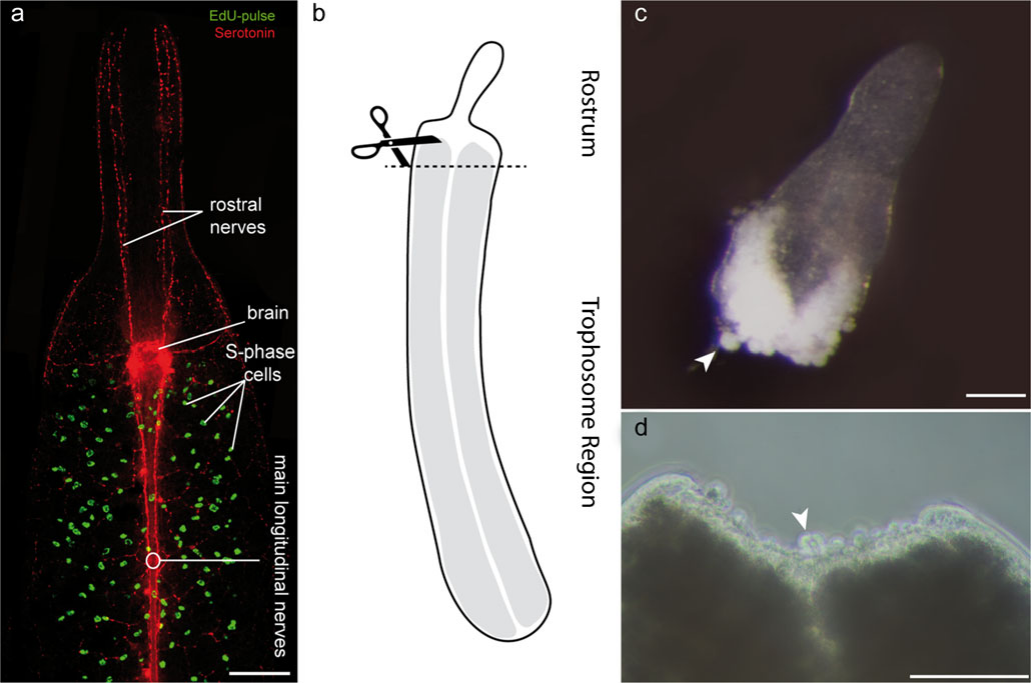
**a** Fluorescently stained, EdU-pulse-labeled *Paracatenula galateia*. EdU-labeled S-phase cells are seen in *green* and antibody-stained serotonergic nerves are seen in *red*. Structures such as the brain, S-phase cells and nerves, which are of special importance for this study, are labeled. **b** Representation of a *P. galateia*. The *scissors* and *dashed line* indicate the position of amputation. **c** Rostral fragment under incident light directly after amputation. Note the single bacteriocytes leaking out of the open wound (*arrowhead*). **d** Interference contrast image of a trophosome region fragment directly after amputation (*arrowhead* spherical cells leaking out of the wound). *Bars* 100 μm

In intact *P. galateia* subjected to a 30-min EdU-pulse (no chase), S-phase neoblasts were detected exclusively in the body region posterior to the brain. The rostrum was always devoid of S-phase neoblasts (Fig. 1a). In this study, freshly collected flatworms were subjected to a 30-min EdU-pulse before the rostrum was transversally amputated (for the cutting plane, see Fig. 1b). The fate of neoblasts that had incorporated EdU during their S-phase was traced later. Additionally, a 30-min pulse with BrdU was applied after various chase/regeneration times, shortly before the experiment was stopped and the animals were fixed. This BrdU-pulse after different time intervals was used to detect proliferating cells during later stages of the regeneration process. Subsequently, EdU, BrdU and serotonergic nerves were fluorescently labeled and then visualized by using a confocal laser scanning microscope.

The amputation resulted in an anterior fragment (rostral fragment; Fig. 1c) and a posterior fragment (trophosome fragment; Fig. 1d). Immediately after cutting, we often found single bacteriocytes emerging from the cutting wound of both fragments (arrowheads in Fig. 1c, d). Within the following 16 days, we documented the processes of rostrum regeneration of the anteriorly regenerating trophosome fragments. A trophosome regrowth of the posteriorly regenerating rostrum fragments was never observed within the limited observation period.

 Here, we first describe a number of steps in the rostrum regeneration process including proliferation and migration patterns of neoblasts, blastema formation and the behavior of the worms.

### Rostrum regeneration of trophosome region fragments

**Fig. 2:**
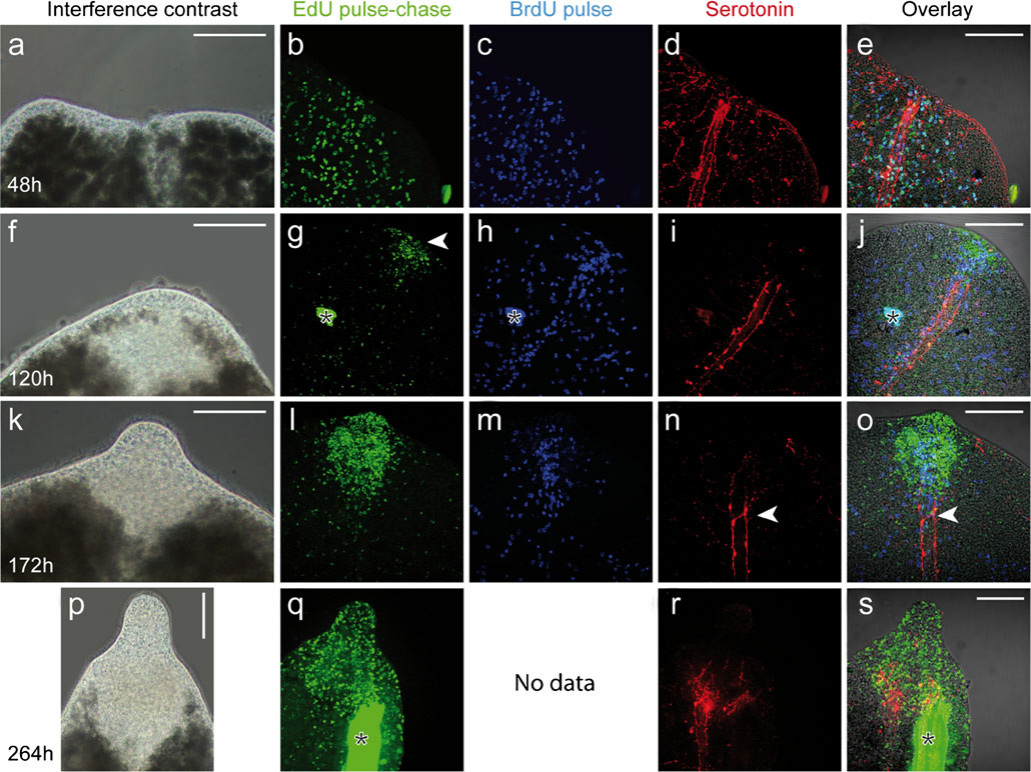
Interference contrast and fluorescence micrographs of various stages of rostrum regeneration of *P. galateia*. Each *horizontal column* of the images belongs to the same stage. The time of regeneration (48 h, 120 h, 172 h and 264 h) is indicated *far left*. *Top* Type of image, incubation, or staining. As indicated by the *color* of the *font*, EdU label is shown in *green*, BrdU label in *blue* and serotonin in *red*. *Far right* Merged view (*Overlay*) of the three fluorescence images *left* plus the interference contrast image (*arrowheads* important events in the regeneration process as described in text, *stars* image artifacts). *Bars* 100 μm

Whereas intact *P. galateia* showed directed forward and backward movement, the freshly decapitated trophosome region fragments temporarily rolled up. After a few seconds, they slowly relaxed and started an idly forward movement. Sometimes, peristaltic contractions of the fragments were observed. Similar behavior has been observed from intact worms as a sign of stress, e.g., after magnesium chloride treatment or freshwater shock. After about 6-12 h, the decapitated fragments became immobile (*n*≥30). The cutting wound was initially reduced in size, possibly by the constriction of circular muscles and had closed within 48 h by the flattening of surrounding epidermis cells (Fig. 2a). In this stage, the bacteriocytes were distributed up to the anterior tip of the worms. EdU and also BrdU labels were evenly distributed in the fragments and almost 60% of the cells showed both labels (Fig. 2b, c, e; *n*=5). This indicated that either the S-phase of the EdU-pulse-labeled neoblasts was still progressing after the 48-h chase, or that some EdU-labeled cells had completed their cell cycle and reached a second S-phase in which they were labeled with BrdU. Serotonin staining of the 48-h anterior regenerates showed longitudinal nerves ending blindly in the wound area and a network of fine subepidermal nerves (Fig. 2d, e; *n*=5).

Five days (120 h) after the amputation, the trophosome region fragments slowly started to move in a forward direction. The bacteriocytes did not reach the anterior end and rostrum regeneration had started (Fig. 2f). A strong aggregation of EdU-pulse-chase-labeled cells was found in the symbiont-free regenerating area and labeled cells were found to be almost evenly distributed in the remainder of the trophosome region, albeit at a far lower density than at the beginning of the experiment (Fig. 2g; *n*=3). In the area of regeneration, we also detected a massive cluster of BrdU-labeled S-phase cells indicating proliferation within this tissue (Fig. 2h; *n*=3). These proliferating S-phase cells were most probably neoblasts forming a regeneration blastema (accumulation of undifferentiated cells at the wound site covered by an epithelium). A further clustering of BrdU-labeled cells was found along the major longitudinal nerve cords, whereas the rest of the body showed a lower density and an even distribution of such cells (Fig. 2h, j). The major longitudinal nerves were still found to end blindly but did not reach the anterior end because of the epigrowth of the blastema (Fig. 2i). After a 120-h chase, EdU/BrdU double-labeled cells were more difficult to detect since the EdU signal intensity was weakened probably by dilution of the label in recent mitoses. Nevertheless, many cells, especially those aggregated in the blastema (site of rostrum regeneration) showed light-blue staining indicating the EdU/BrdU double-label (Fig. 2j; *n*=3).

**Fig. 3:**
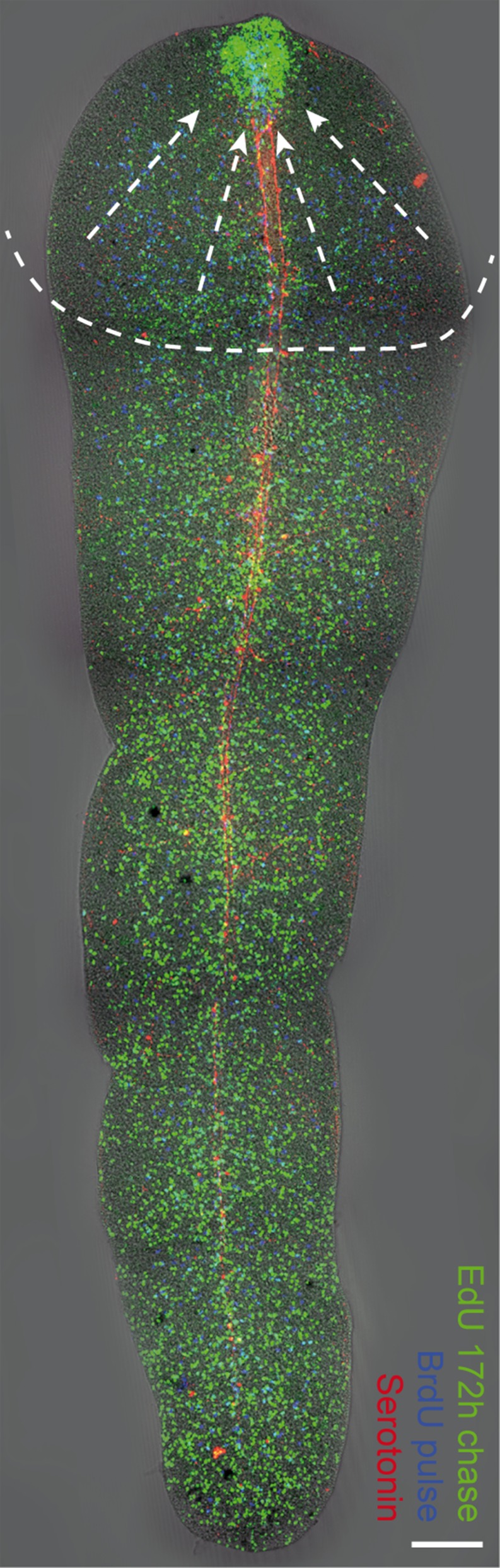
*P. galateia* trophosome region fragment 172 h after decapitation. Cells labeled by a EdU pulse and 172-h chase (*green*) are strongly aggregated in the regenerating rostrum area. The labeled cells in the anterior region of the worm have been recruited to the wound area to contribute to the regeneration process (*arrows* and *dashed lines* roughly indicate the sphere of influence). The EdU-labeled cells show an even distribution in the posterior region. Cells labeled after a BrdU pulse but no chase (*blue*) are evenly distributed over the worm's body and slightly aggregated in the regenerating rostrum. The light-blue staining indicates EdU/BrdU double-labeled cells. The staining of serotonergic nerves is shown in *red*. *Bar* 100 μm

The next step in the regeneration process, around 172 h after decapitation, was the outgrowth of the tip of the rostrum (Fig. 2k). By this time, all worms had restored their normal locomotion. The regenerating rostrum was completely free of symbionts and EdU-labeled cells were found to be considerably more aggregated in the blastema (Fig. 2l; *n*=5). In the anteriormost 500-600 μm of the trophosome worms showed a clearly lowered density of EdU-labeled cells, which reflected the recruitment of the EdU-pulse-chase-labeled cells into the site of regeneration (Fig. 3). In the same worms, the density of BrdU-labeled cells in the blastema was also increased compared with the remainder of the worm's body, which showed an even distribution of BrdU-labeled S-phase cells (Fig. 2m; *n*=5). Double-labeled cells were found in the center of the blastema (Fig. 2o). By this time, the nerves showed the first clear indications of reorganization. A prominent commissure was visible at the anterior end of the major longitudinal nerves and tiny nerve branches extended further in an anterior direction (arrowheads in Fig. 2n, o), possibly representing the rostral nerves found in the rostrum of intact *P. galateia* (cf. Fig. 1a).

After around 264 h of regeneration, the rostrum had regained the typical shoulder-shaped transition between the rostrum and trophosome region (Fig. 2p). A large proportion of the cells constituting the newly formed rostrum showed EdU labeling (Fig. 2q; *n*=3). The staining of the BrdU-labeled S-phase cells was unsuccessful in all of the specimens that we observed; this was traced to a faulty batch of BrdU. On the boundary of the trophosome and rostrum, the major longitudinal nerves terminated in a commissure with an intense accumulation of serotonergic nerves indicating the advanced state of brain regeneration (Fig. 2r, s; *n*=3).

**Fig. 4:**
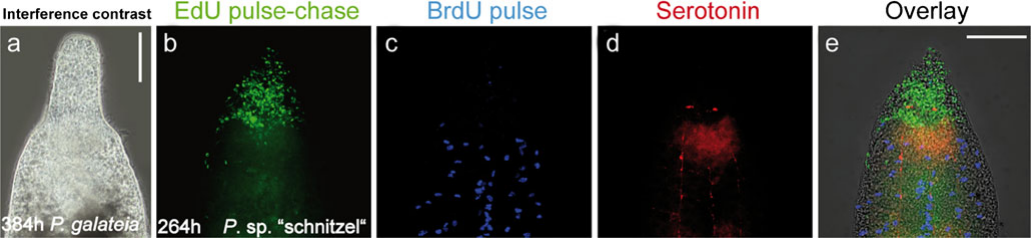
**a** Interference contrast image of *P. galateia* after 384 h of rostrum regeneration. **b–e** Fluorescent images of *Paracatenula* sp. “schnitzel” after 264 h of rostrum regeneration. **b** EdU-pulse with 264-h chase (*green*). **c** BrdU pulse-staining (*blue*). **d** Staining of serotonergic nerves (*red*). **e** Merged images (*Overlay*). *Bars* 100 μm

In *P. galateia*, rostrum regeneration appeared to be complete after about 384 h (Fig. 4a). No difference in the behavior of these regenerates compared with the non-amputated control animal could be observed (*n*=4). For technical reasons, the contribution and fate of neoblasts in the final step of rostrum regeneration could not be analyzed in *P. galateia*: none of the *Paracatenula* species has to date been successfully cultured and thus, all experiments were carried out on freshly collected *P. galateia*; in the limited time frame of 20 days on Carrie Bow Cay Island (Belize), the long-term (chase ≥10 days) regeneration experiments including the pulse-chase-pulse labeling were not successful. Alternatively, we studied this final step in another, as yet undescribed species of the genus, called *Paracatenula* sp. “schnitzel”. The regeneration process followed the same pattern as described for *P. galateia* but was completed slightly faster after around 264 h. The pulse-chase EdU label in *P. schnitzel* was highly aggregated in the regenerated rostrum (Fig. 4b; *n*=3) comparable to the label observed in *P. galateia*. The BrdU-labeled S-phase cells were restricted to the region posterior to the brain and no signals were detected in the rostrum (Fig. 4c, e; *n*=3). This indicated the disappearance of the blastema and the restoration of the original condition in which neoblasts were restricted to the body region posterior to the brain. In addition to the regenerated brain at the boundary of the trophosome, the serotonin staining showed longitudinal nerves (Fig. 4d, e; *n*=3). The rostral nerves were either much more delicate than those seen in *P. galateia* and therefore not so easy to see, or they had still not completely developed (cf. Fig. 1a).

### Pattern of neoblast proliferation and migration in amputated rostrum fragments

**Fig. 5:**
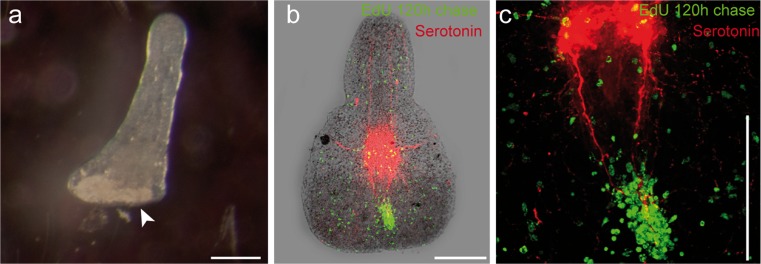
Posterior regenerating rostrum fragments. **a** Rostrum fragment 48 h after amputation under incident light. The wound on the posterior end is almost closed by the flattening of surrounding epidermal cells (*arrowhead*). **b** Rostral fragment labeled by an EdU-pulse with 120-h chase. The EdU-labeled cells (*green*) are evenly distributed in all areas of the fragment but a massive aggregation of cells encapsulating the posterior tip of the longitudinal main nerves (*red*) is also seen. **c** Higher magnification of the labeled cells encapsulating the tip of the nerves. *Bars* 100 μm

Compared with intact worms, the freshly amputated rostrum fragments showed no obvious change in their behavior besides increased speed and agility. Posterior regeneration of rostrum fragments, like the regeneration of the anterior trophosome fragments, started with the reduction of the wound size by constriction of the ring musculature and wound closure within 48 h, including a flattening of surrounding epidermal cells (Fig. 5a, *arrowhead*). Within the following 16 days, all rostrum fragments died without showing any further signs of posterior growth/regeneration. Two rostral fragments labeled by an EdU-pulse with a 120-h chase could be fixed and analyzed. In both, the EdU-labeled cells had migrated into the rostrum and several EdU-labeled cells showed overlapping serotonin staining. Whereas one of the rostral fragments showed an even distribution of EdU label, an aggregation of cells encapsulating the posterior tip of the longitudinal main nerves was observed in the other (Fig. 5b, c).

## Discussion

### Morphallaxis and behavior

In *P. galateia*, a predominantly asexually reproducing catenulid, rostrum amputation is always followed by morphallactic processes leading to wound closure. This process is probably a plesiomorphy of all flatworms, since the same pattern has been observed in various taxa (for reviews, see Egger et al. 2006, 2007; Moraczewski 1977). Moreover, the behavior that both fragment types (anterior and posterior fragments) show after amputation and during regeneration is similar to that described in other flatworms. The first reaction to decapitation is invariably an escape-reaction-like movement backward or a coiling up. After the first “shock effect”, fragments containing the brain often continue moving rapidly, whereas brainless fragments remain relatively stationary during regeneration until the brain is restored (Moraczewski 1977; Reddien and Sanchez Alvarado 2004).

### Proliferating cells in the blastema

The cells proliferating during regeneration have been characterized as neoblasts in various flatworms (Egger et al. 2009; Moraczewski 1977; Newmark and Sanchez Alvarado 2000). This is the reason that we have also assumed that all the proliferating cells in the blastema of *P. galateia* are neoblasts. With respect to intact *P. galateia*, we have shown that all S-phase cells in the worms have typical neoblast morphology (Dirks et al. 2012). Some of these features are chromatoid bodies, a small size, an extremely high nucleo-cytoplasmic ratio and a characteristic heterochromatin pattern (Auladell et al. 1993; Fernandéz-Taboada et al. 2010; Hori 1982; Kotaja and Sassone-Corsi 2007). Nevertheless, we cannot exclude that, during the regeneration of *P. galateia*, other cell types regain the capability to proliferate. In this study, a more detailed investigation of the blastema has not been possible because of the lack of further samples of the non-culturable animals. Future studies will have to address these questions by analyzing the ultrastructure of the cells proliferating in the different stages of the blastema.

The detailed proliferation pattern of neoblasts during the regeneration process has been studied in other flatworms. In *M. lignano*, by 24 h after amputation of the tail plate, the wound is closed and a blastema has been formed. Inside this blastema, the neoblasts (labeling of S-phase and mitoses) rapidly proliferate until the cells start progressively to differentiate at about 72 h after amputation, when regeneration is nearly completed (Egger et al. 2009). In *P. galateia*, we see a comparable but clearly slower progression of proliferation and differentiation during regeneration. We have detected three stages: (1) wound closure, (2) blastema formation and proliferation and finally (3) the dissolution of the blastema and cell differentiation. To increase our knowledge about the regeneration process in *P. galateia*, further intermediate stages should be investigated in whole mounts and sections.

### Slow regeneration

In the species *Paracatenula* cf. *polyhymnia*, the rostrum regeneration process takes only 48-72 h (Dirks et al. 2012). Moraczewski (1977) has described that one *Catenula* species needs about 60 h for rostrum regeneration and Palmberg (1991) has shown that *Microstomum lineare* regenerates its rostrum completely in only 45 h. The time that *P. galateia* needs to regenerate the complete rostrum, namely 2 weeks, is lengthy in comparison with the above-mentioned species. The most obvious difference between *P. galateia* and these other three species is in their size. *P.* cf. *polyhymnia* has a roundish cross section and diameter of 50-60 μm (Leisch et al. 2011). Moreover, *Catenula* sp. and *M. lineare* are roundish worms with diameters of less than 100 μm. *P. galateia* is, in contrast, a larger species with a dorsoventrally flattened body and a width of 250-300 μm. Thus, the amount of tissue that *P. galateia* has to regenerate is far greater than those in the other species; this might be one reason for the slower regeneration. On the other hand, the quantity of proliferating cells in *P. galateia* is accordingly higher.

Additionally, EdU-chase-BrdU experiments indicate that the cell cycle (S-phase, in particular) takes more than 48 h in total in flatworms. Preliminary experiments with non-amputated *P. galateia* in which we have used nocodazole to accumulate pulse-labeled cells in the mitosis point to the length of S-phase and G2-phase as being 48-72 h (own unpublished data). This is exceptionally slow compared with other flatworms. In *M. lineare*, Palmberg (1990) has detected partly differentiated cells as early as 6 h after S-phase labeling. For *P*. cf. *polyhymnia* and *Catenula* sp., no data on the length of the cell cycle are available. Careful measurements of the cell cycle length in *Macrostomum lignano* have revealed that it passes through both S-phase and G2-phase within maximal 24 h (and probably even faster; Nimeth et al. 2004). As has been shown in various animal systems, in addition to the known checkpoints at which the eukaryotic cell cycle can be arrested, certain conditions such as the lack of nutrients can slow down the progress of different parts of the cell cycle, including the S-phase (Alexiades and Cepko 1996; Paulovich and Hartwell 1995; Steinemann 1980). Not only is *P. galateia* possibly traumatized at the loss of its head but it is also likely to be starving under the present culture conditions. The worms do not have a mouth or a gut and thus totally depend on their bacterial symbionts for nutrition (Gruber-Vodicka et al. 2011). To date, none of the known *Paracatenula* species has been successfully cultivated, probably because the host and symbiont eventually starve to death. Therefore, a starvation-induced progressive “slow-down” of the cell cycle might be possible. The smaller species *P*. cf. *polyhymnia*, which regenerates its rostrum within 48-72 h (Dirks et al. 2012) might not be affected by this slow-down, if the worms require a period within which to switch to starvation metabolism. Regeneration and labeling experiments with *P*. cf. *polyhymnia* and further studies on the cell cycle length of *P galateia* should help to answer these questions. Until cultivation of the symbiosis is possible, resolution of these questions will remain difficult.

### No posterior regeneration

Within 16 days of observation of the *P. galateia* rostrum fragments, neither the growth of the symbiont-housing trophosome tissue nor of the symbiont-free “tail” (which is present in other *Paracatenula* species; see Dirks et al. 2011) occurs. However, we find it hard to believe that these worms are incapable of regenerating posteriorly, since no other example of animals that can regenerate anteriorly but not posteriorly are known (for a review, see Egger et al. 2007). Here, we provide four hypothetical explanations for the absence of posterior regeneration from rostrum fragments. (1) The number of neoblasts in the rostrum fragments is too small to accomplish posterior regeneration. The rostrum is devoid of neoblasts (see Fig. 1a) and thus, anterior fragments only contain a small number of these cells. Since neoblasts are the major (presumably only) drivers of regeneration, their near absence might lead to a slow-down or even to an incapability to regenerate. Moreover, *Macrostomum* and triclads lack neoblasts in their most anterior region. When cut in front of the photoreceptors, the anterior fragment is unable to regenerate because of the lack of neoblasts (for a review, see Reddien and Sanchez Alvarado 2004). (2) In flatworm species from various taxa, the regeneration of any body part has been observed to require certain parts of, for example, the pharynx in the case of *M. lignano* or the intestine in the case of *Catenula* sp. (Egger et al. 2006, 2007; Moraczewski 1977). In *Paracatenula*, these endodermal organs are lacking and have been functionally replaced by the trophosome. To date, the germ layer from which the trophosome derives remains unknown. If it originates from the endoderm and is a modification of the pharynx or gut, the inability of *P. galateia* to regenerate posteriorly may have its cause in the lack of this organ. (3) The loss of almost the whole trophosome should result in insufficient host nutrition by the symbionts. This starvation might lead to a slow-down or even arrest in the neoblast cell cycle preventing regeneration and the growth of the eukaryotic tissue. In other flatworms, starvation has even been shown to lead to the shrinkage and degeneration of certain organs (Baguna 1976; Baguna and Romero 1981; Nimeth et al. 2004). The finding that no attempt at cultivation of any *Paracatenula* species has been successful so far might point to the inappropriate nutrition (culture conditions) of one or both symbiotic partners. (4) Finally, the trophosome region consists solely in the symbiont and houses bacteriocytes surrounded by an epidermis. If the bacterial symbionts do not grow (proliferate), the regeneration and growth of the trophosome region would be a waste of energy and resources for the host.

For all these reasons, the animals are extremely unlikely to be able to regenerate posterior regions under natural nutrition conditions. If the trophosome region will not grow, repeated paratomy would lead to ever smaller fragments and eventually to the death of all fragments.

### Regeneration of a symbiotic metazoan

Since the studies of Thomas Hunt Morgan (1905), it was initially speculated and is today well known that a gradient of morphogens is important for re-establishing axes and the identities of tissues in regenerating flatworms. To form a gradient along an axis, a minimal tissue size (or cell number) is required (for a review of regeneration gradients in planarians, see Adell et al. 2010). In *P. galateia*, we have not determined the smallest trophosome region fragments capable of regenerating a rostrum but have rather found that small fragments with an anterioposterior length of 0.5 mm regrow a rostrum on only the anterior side (Dirks et al. 2012). Despite almost 40% of the trophosome region of *P. galateia* being made up by bacterial symbionts (Gruber-Vodicka et al. 2011), this high symbiont/host ratio apparently does not negatively influence the morphogenic gradients necessary to establish its axis formation during regeneration. Future studies will reveal further details concerning gradient formation during the regeneration of lost body parts in *P. galateia*.

With the exception of the peritrich ciliate *Zoothamnium niveum*, no chemosynthetic symbioses or isolated symbionts have been successfully cultivated (Dubilier et al. 2008; Rinke et al. 2007). Although we have made good progress in keeping *P. galateia* alive for several weeks, permanent cultivation is not possible at the moment. Therefore, the carrying out of experimental studies on these fragile and rare organisms is a challenge. Nevertheless, an understanding of developmental processes and host-symbiont interactions in this exceptional tight animal-bacteria symbiosis model of *P. galateia* is of great importance for the field of symbiosis research.
